# “Persisters”: Survival at the Cellular Level

**DOI:** 10.1371/journal.ppat.1002121

**Published:** 2011-07-28

**Authors:** Clinton C. Dawson, Chaidan Intapa, Mary Ann Jabra-Rizk

**Affiliations:** 1 Department of Oncology and Diagnostic Sciences, Dental School, University of Maryland, Baltimore, Maryland, United States of America; 2 Department of Microbiology and Immunology, School of Medicine, University of Maryland, Baltimore, Maryland, United States of America; University of California San Francisco, United States of America

## Introduction

Rather than being slowly eroded and destroyed, countless numbers of varied forms of life adapt to the diverse aspects of an ever changing environment. However, the amount of variation is maintained at a practical optimum, as too much variation would make the population ill-adapted in a stable environment, while too little variation would render it unable to adapt to environmental stresses. This principle is perhaps well exemplified by a phenomenon described for microbial cells termed “persistence” where in the face of antibiotics bacterial populations avoid extinction by harboring a subpopulation of drug-insensitive dormant cells. Although this phenomenon poses a major obstacle for the treatment of infectious diseases, persistence has been underappreciated for some time as a mechanism for bacteria to evade antibiotics. But the mechanisms of bacterial persistence are becoming clearer and so are ways to combat them. This article highlights the phenomenon of survival and persistence in cells as diverse as microbial and human and summarizes the recent advances that have taken us one step closer to understanding what persistence is all about.

## Microbial “Persister” Cells

**Figure 1:**
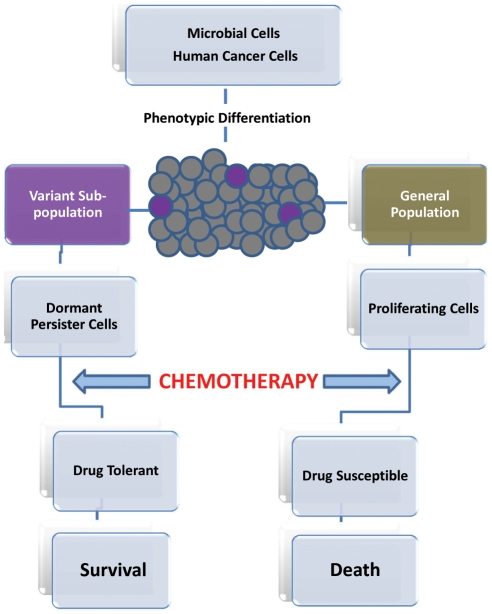
Progression of persister cell development and enhanced drug tolerance.

In the early 1940s, it was only appropriate for Joseph Bigger to refer to a small subpopulation of bacterial cells that survived killing by penicillin, as “persisters” [1]. These small numbers of cells were then proposed to be dormant and nongrowing phenotypic variants of the general cell population [2], [3]. This theory of “persisters” has since been established in various bacterial populations. However, more recently the existence of a small cell subpopulation that can remain viable at high concentrations of an antifungal agent was described for the fungal pathogen *Candida albicans* [4]–[6]. Therefore, it has become clear that the ability to avoid killing is a key characteristic common to all microbial persisters that are not mutants, but rather phenotypic variants that can survive antimicrobial treatment. However, unlike drug resistance, drug tolerance appears to be a transient and reversible physiological state in a small subpopulation of genetically identical cells [7], [8]. When the antimicrobial agent is removed, these persisting microbial cells not only resume growth, but their progeny is sensitive to the antimicrobial agent (Figure 1) [7], [8].

## Formation of Drug Tolerant Persisters

Persisters have been described to arise spontaneously on the basis of random stochastic events [2], [3], [9]. Stochasticity can be advantageous in providing flexibility for the cells to adapt to fluctuating environments and sudden stresses and, therefore, stochastic mechanisms are thought to lead to the emergence of phenotypically distinct subgroups within isogenic cell populations [9]. However, defined inducible mechanisms have been recently identified to play a role in persister cell formation [10].

### Quiescence and Biofilms

Recent findings from studies examining the rate of bacterial persister-cell formation over time showed that the highest frequency (∼1%) occurs in the nongrowing stationary phase [5]. Interestingly, when the culture was kept in early exponential phase by repeated regrowth, persister cells disappeared, indicating that persisters are preformed rather than produced in response to stress [5]. Furthermore, gene expression studies demonstrated the downregulation of transcription of genes involved in energy production and nonessential functions concomitant with upregulation in genes associated with cellular arrest [2]. These findings are consistent with the description of persister cells as being dormant, a transient state of existence that would impede the ability of drugs to corrupt their target molecules in the microbial cell [7]. In that respect, entry into quiescence is advantageous; however, it is more beneficial for a cell to be a dividing cell than a dormant cell. Therefore, it is more likely that the optimal cell strategy is not to enter into persistence, suggesting that the persister state is an altruistic behavior to ensure the continuation of the population.

The simplest strategy to trigger entry into dormancy would be to overproduce proteins or toxins that inhibit cellular processes and growth [3], [11]. One such identified factor is the high persistence gene *hipA*, which encodes a toxin (HipA) that inhibits translation in *Escherichia coli*. This toxin was identified to be implicated in forming persisters because its overexpression increased the frequency of persistence by 10,000-fold and resulted in drug tolerance [12]. HipA is normally neutralized by HipB, a transcription repressor that counteracts HipA by attaching to it preventing it from shutting down protein production and, therefore, *hipBA* has been categorized as a toxin/antitoxin (TA) module [11], [12]. Recently, through extensive studies including structural analyses, Schumacher et al. [12] identified HipA to be a protein kinase that phosphorylates the translation factor EF-Tu. These findings demonstrating that HipA bound the EF-Tu peptide supported the hypothesis that HipA mediates persistence by phosphorylating one or more target proteins. On the basis of these new insights into the mechanisms by which HipA mediates persistence, the authors suggested that inhibitors that specifically target the substrate-binding sites of HipA may prove effective against persistence.

Perhaps, the best defined mechanism by which persister bacterial cells arise comes from the fact that DNA damage induces one or more components of the protective SOS stress response, a signaling pathway that upregulates DNA repair functions [13]. Specifically, in *E. coli*, exposure to a DNA-damaging antibiotic triggered the gene encoding a small membrane-acting peptide TisB, which decreases proton motive force and ATP levels suggesting that TisB protein may induce dormancy by shutting down cell metabolism [14]. These speculations were substantiated by the findings demonstrating that deletion of the *tisB* gene resulted in decreased frequency of persisters tolerant to DNA-damaging antibiotic [14]. Interestingly, although overexpression of *tisB* resulted in cell death, minor overproduction of the peptide induced persister formation suggesting that induction of TisB is involved in the production of multidrug tolerant cells, in turn identifying *tisB* as a persister gene [14]. Combined, these observations are in accordance with the perception that dormancy and SOS response represent strategies of cell survival.

Persister cells are highly enriched in biofilms, which are complex and highly organized surface-attached communities of microbes embedded in a polymeric matrix [8], [15]. Biofilms form on abiotic surfaces and host tissue and are responsible for infections of indwelling medical devices. It is estimated that over 65% of all infections are biofilm-associated, which tend to be difficult to eradicate because of enhanced resistance to antimicrobials [2], [16]. The biofilm environment is advantageous to the microbial populations, however, when nutrients become limited metabolic dormancy becomes the viable option [8]. In a clinical setting, when most cells in a biofilm are readily killed by low concentrations of antibiotics, the small metabolically dormant phenotypes progress to become tolerant persister cells. By virtue of their dormancy, this subpopulation of cells confer benefits to the general cell population and are in turn responsible for the high tolerance of bacterial biofilms to antimicrobial agents [3], [7].

Persisters are formed by all bacterial species studied and are present at 0.1%–1% in the biofilms of *Pseudomonas aeruginosa*, *E. coli*, and *Staphylococcus aureus* [17]. Recently, the existence of a small cell subpopulation that can remain viable at high concentrations of antifungal agent has been described in fungal biofilms, specifically for the human pathogen *C. albicans* [4], [6]. Clinically, candidal infections may resolve upon antifungal therapy but often remain recalcitrant to treatment. In a recent study, on the basis of tolerance to high doses of an antifungal agent, invariably all *C. albicans* isolates recovered from nonresolving infections appeared to be high-persister variants. Similar to bacteria, *C. albicans* forms adherent biofilms, which are essentially recalcitrant to antifungals [16], [18]. The mechanism of *C. albicans* biofilm antifungal resistance remains largely unknown; however, biofilms have been described to exhibit a biphasic killing pattern in response to antimicrobial agents, indicating that a subpopulation of highly tolerant cells existed [6], [7]. Interestingly, reinoculation of surviving cells produced a new biofilm with a new subpopulation of persisters. These observations suggest that *C. albicans* persisters, analogous to their bacterial counterparts, are not mutants but phenotypic variants and that attachment to a surface is what initiates dormancy that leads to the formation of persisters [5], [6].

## Cancer Persister Cells

Similar to the obstacle in treatment of patients that develop resistance to antimicrobials, acquisition of resistance to anticancer drugs is a major problem in cancer therapy. Most treatments, even ones that work, fail over time because tumor cells become resistant. Different mechanisms of resistance have been described for cancer cells such as modification of drug target and active extrusion of drugs by efflux pumps and, therefore, it was largely assumed that random gene changes confer resistance to drugs [19]. However, this does not explain an increasingly observed phenomenon in cancer chemotherapy; “retreatment response” [20], [21]. In this model, it is proposed that once a small number of cells that survive exposure to drugs that killed the majority of the cells are given a “drug holiday,” they eventually regain their sensitivity to the drug [22]. These observations indicate that acquired resistance to cancer drugs may not necessarily result from stable genetic mutations but may also involve a reversible “drug-tolerant” state [22], [23].

In a recent study by Sharma et al. [22], drug-sensitive cells were treated with antitumor drugs at concentrations exceeding 100 times the established IC_50_ values. Following three rounds of 72-h treatments, the authors consistently detected a small subpopulation of reversibly “drug-tolerant” cells demonstrating >100-fold reduced drug sensitivity. Further analyses demonstrated that these cells maintained viability via engagement of insulin-like growth factor 1 (IGF1) receptor signaling and an altered chromatin state and treatment with IGF1 receptor inhibitors or chromatin-modifying agents selectively ablated the drug-tolerant subpopulation

Cancer-initiating cells are proposed as a potential resistant subpopulation because of their ability to escape the effect of drug treatment by becoming quiescent [24]. This transient drug-tolerant state could provide a mechanism that allows a small subpopulation of tumor cells to withstand an initial destructive attack of drug to enable their survival, until more permanent resistance mechanisms can be established [22]. Intriguingly, this transient ability to endure anticancer drugs was recently reported to be highly reminiscent of the drug-tolerant microbial “persister” subpopulations [22], [25]. In that sense, it is plausible to regard slow-growing cancerous cells in highly proliferating tumors to be analogous to microbial persister cells in biofilm (Figure 1).

## Conclusion and Future Directions

Whether microbial or human in nature, it appears that cells have evolved analogous redundant strategies where the function of survival is assigned to a small dormant subpopulation of cells within a more rapidly proliferating population. With most of the currently available chemotherapeutic agents targeting exponentially growing cells, our therapeutic arsenal is ineffective in eradicating these dormant persister cells. Coupled with the increasing emergence of drug resistance and failure of therapies despite our medical advances, it has become critical to develop novel classes of drugs. The prospect that persisters are responsible for the persistence of chronic infections and, more gravely, recalcitrance of disseminating cancers have identified these culprit cells as viable targets for new therapies. However, such discoveries rely heavily on the depth of our understanding the nature of these intriguing cells, which would provide us with fundamental insights into the mechanisms involved in the development of drug tolerance. Inopportunely, their transient nature and low abundance, has impeded experimental advancements to elucidate the dynamics of the formation of these specialized cells that neither die nor grow. Nevertheless, the recent unearthing of an inherent tactical approach shared by diverse cellular insurgents will undoubtedly herald a new era of research into the new field of “persisters.”
